# Obesity and its implications for COVID-19 pandemic in South Africa

**DOI:** 10.4102/sajid.v36i1.228

**Published:** 2021-04-20

**Authors:** Fiona A. van Vollenstee, Maria-Teresa van der Merwe

**Affiliations:** 1 School of Medicine, Internal Medicine, Faculty of Health Sciences, University of Pretoria, Pretoria, South Africa; 2 Centre of Excellence of Metabolic Medicine and Surgery (CEMMS) South Africa, School of Medicine, University of Pretoria, Pretoria, South Africa

To the Editor

Dear Madam, Sir,

South Africa has been categorised within the top 10 countries with the highest obesity prevalence, with population projections of 47.7% for females, 23.3% for males and 22.1% for children (5–19 years old) to be obese (body mass index [BMI] > 30 kg/m^2^) by 2025.^1^

Considering the coronavirus disease 2019 (COVID-19) pandemic and predisposed risk factors, literature recently emphasised the need for increased vigilance, priority on detection and testing, as well as prompt and aggressive therapy for patients with obesity.^2,3^

Obesity is a non-communicable disease associated with variety of a comorbidities, which includes: (1) metabolic diseases: type 2 diabetes mellitus (T2DM), metabolic associated fatty liver disease (non-alcoholic fatty liver disease [NAFLD] or non-alcoholic steatohepatitis [NASH]), dyslipidaemia and chronic inflammation, (2) respiratory diseases: chronic hypoventilation, sleep apnoea, chronic obstructive pulmonary disease and (3) cardiovascular diseases: hypertension, pulmonary embolism and coronary heart disease.^2,3,4,5^ Age < 60 years and co-morbidities linked to obesity are powerful predictors of hospitalisation length of stay, intensive care unit (ICU) admissions, higher morbidity and disease progression because of acute respiratory syndrome coronavirus-2 (COVID-19), despite correcting for comorbidities.^3,4,5,6^

Weight loss achieved with health behavioural changes could be 3% – 5% of body weight, however long-term adherence to reduced caloric intake even with optimal medical nutritional therapy could be difficult to achieve.

Pharmacotherapy has been recommended to patients with BMI > 30 kg/m^2^ struggling with adiposity-related complications,^7^ but bariatric surgery is still recognised as the most clinically, cost-efficient and long-term sustainable treatment option for patients with complex obesity.^7,8^ Bariatric surgery eligibility in South Africa is more stringent than international guidelines, where a BMI > 47 kg/m^2^ would qualify a patient or a BMI > 35 kg/m^2^ with two or more obesity-related comorbidities compared with international guidelines where a BMI > 40 kg/m^2^ and a BMI > 35 kg/m^2^ with one obesity related comorbidity would be eligible.^7^

Bariatric surgery is known to be associated with micro- and macro-nutritional deficiencies, but with a comprehensive and experienced multidisciplinary team providing an individualised multi-therapeutic approach, these side-effects should be well managed.^8^

At the University of Pretoria, under the Centre of Excellence of Metabolic Medicine and Surgery (CEMMS), patients with obesity and associated comorbidities have been monitored before and after bariatric surgery. All patients who received surgery between January 2014 and December 2019 were considered. From the data table (Table 1), clinical and biochemical parameters were assessed at baseline and 12 months post-surgery and resolution of comorbidities were assessed at 1 and 3 years post-surgery? Significant findings were observed at 12 months post-surgery: (1) reduction across all clinical parameters including actual weight and BMI (2) reduction in the number of comorbidities (6.79 to 1.35), improvement in glucose control and reduction in dyslipidaemia (Table 1). At 3 years post-surgery follow-up sustained reduction in comorbidities was observed. These data are in line with findings from international bariatric centres and ongoing research, including the Swedish Obese Subjects Study.^9^

**TABLE 1:** Clinical and biochemical outcomes and remission of comorbidities for patients with obesity receiving bariatric surgery at baseline, 12-months and 3-years, respectively, after surgery.

Clinical parameters	All patients at baseline (*n* = 1179)	All patients 12 months post-surgery (*n* = 768)	*p*	Patients at baseline (%) (*n* = 1035)	Patients at 1-years post-surgery (%) (*n* = 756)	Patients at 3-years post-surgery (%) (*n* = 276)
Weight (kg)	131.70 ± 0.83	86.26 ± 0.60	***	-	-	-
BMI (kg/m^2^)	46.61 ± 0.24	30.25 ± 0.17	***	-	-	-
Neck circumference (cm)	47.62 ± 0.19	35.67 ± 0.13	***	-	-	-
Waist circumference (cm)	130.19 ± 0.55	98.48 ± 0.48	***	-	-	-
Hip circumference (cm)	135.27 ± 0.50	106.27 ± 0.42	***	-	-	-
BP (S) RR < 130 mmHg	145.37 ± 0.47	125.40 ± 0.57	***	-	-	-
BP (D) < 85 mmHg	86.27 ± 0.34	75.36 ± 0.39	***	-	-	-
Heart rate (pulse/min)	79.32 ± 0.37	63.39 ± 0.39	***	-	-	-
Number of comorbidities†	6.79 ± 0.06	1.35 ± 0.06	***	-	-	-
Biochemical parameter						
F-Glucose (mmol/L) RR: 3.9–6.0	5.64 ± 0.06	4.75 ± 0.03	***	-	-	-
HbA1c (%) RR: 4.0–6.0	5.93 ± 0.04	5.11 ± 0.06	***	-	-	-
F-TG (mmol/L) RR: 0.5–1.6	1.72 ± 0.03	1.10 ± 0.02	***	-	-	-
F-HDL cholesterol (mmol/L) RR: 1.0–1.6	1.18 ± 0.01	1.33 ± 0.01	***	-	-	-
F-LDL cholesterol (mmol/L) RR: 1.6–2.9	3.32 ± 0.03	2.34 ± 0.03	***	-	-	-
GGT (U/L) RR: < 60	36.17 ± 1.02	23.60 ± 1.16	***	-	-	-
ALT (U/L) RR: < 50	32.01 ± 0.64	32.53 ± 1.27	0.69	-	-	-
AST (U/L) RR: < 38	25.68 ± 0.43	28.13 ± 0.74	**	-	-	-
CRP (mg/L) RR: < 5	11.29 ± 0.29	3.27 ± 0.28	***	-	-	-
**Comorbidities†**						
Sleep apnoea	-	-	-	67.6	7.4	7.25
Renal calculi	-	-	-	9.8	2.7	1.1
Pulmonary embolism	-	-	-	3.2	0.4	0.3
Oesophageal reflux or erosions	-	-	-	76.4	19.6	28.7
NAFLD or NASH	-	-	-	76.3	15.9	15.9
Lymphoedema	-	-	-	64.0	13.8	11.2
Insulin resistance	-	-	-	75.2	27.1	30.1
T2DM and IGTT	-	-	-	33.0	5.2	5.4
Idiopathic Intracranial Hypertension	-	-	-	0.7	0.1	0.0
Dyslipidaemia	-	-	-	60.5	22.7	21.4
CVA	-	-	-	0.9	0.1	0.0
Coronary heart disease	-	-	-	3.2	1.1	1.8
Cardiomyopathy	-	-	-	7.0	1.2	0.0
Gallbladder disease	-	-	-	18.7	4.6	2.2
Asthma and COPD	-	-	-	14.1	4.9	5.8

Note: Values expressed in mean ± SEM. Unpaired *t*-test Baseline versus 1- and 3-year post-surgery.

BMI, body mass index; RR, reference range; BP(S), blood pressure systolic; BP(D), blood pressure diastolic; F-glucose fasting glucose; HbA1c, haemoglobin A1c; F-TG, fasting triglyceride; F-HDL, fasting high density lipoproteins; F-LDL, fasting low density lipoproteins; GGT, gamma-glutamyl transferase; ALT, alanine amino transferase; AST, aspartate aminotransferase; CRP, C-reactive protein; NAFLD, non-alcoholic fatty liver disease; NASH, non-alcoholic steatohepatitis; T2DM, type 2 diabetes mellitus; IGTT, impaired glucose tolerance; CVA, cerebrovascular accident; COPD, chronic obstructive pulmonary disease.

* , *p* < 0.05;

** , *p* < 0.01;

*** , *p* < 0.0001.

† , Significant remission of comorbidities within 12 months after surgery and maintained remission for up to 3 years post-surgery.

Possible reasons for severe COVID-19 disease amongst obesity patients include: (1) Angiotensin-converting enzyme 2 (ACE2) is a binding receptor with high affinity for COVID-19, where higher levels of ACE2 ribonucleic acid (RNA) were found in the heart, kidneys, intestinal tract, gallbladder, adipose tissue, testicles and in lungs, which could explain multi-organ dysfunction in patients with severe COVID-19.^3,10^ Patients with obesity carrying excess amount of adipose tissue rich in ACE2 receptors would make them more vulnerable to acquire COVID-19 and possible severe disease progression. (2) Circulation through the hepatic reticular system and activation of Kupffer cells, could explain heightened liver function parameters and hypoalbuminemia observed.^10^ (3) Increased levels of inflammatory markers (d-dimer and c-reactive protein) and expression of pro-inflammatory cytokines (tumour necrosis factor α, interleukin 6, interferon α and β) are considered part of acute inflammatory response adding to disease progression.^3,4,11^ Increased type-2 inflammation is associated with obesity and metabolic syndrome, affecting multiple organs including lungs (parenchyma and bronchi), liver and visceral adipose tissue that could offer a synergistic effect for disease progression.^4,11^ (4) Restricted pulmonary function, residual capacity and reduced lung volumes, as a result of disproportionately high percentage allocation of total body oxygen towards respiratory function, evolve favourably with weight loss following bariatric surgery.^9,11^ Emerging case reports also suggests critical COVID-19 disease is associated with hypercoagulability, which includes pulmonary emboli and micro thrombi.^3^

Similar demographic findings are observed amongst COVID-19 obesity patients compared with infections from other acute viral respiratory pathogens^3^ Considering the experience gained from the Influenza A H1N1 virus (H1N1) epidemic in 2009, the impact of COVID-19 on obesity patients is not surprising, considering the multi-organ failure and damaged immune systems.^3,10,11^ In 2019, New Mexico with an obesity prevalence of 35%, demonstrated a high incidence of H1N1 infections of 46% amongst patients predisposed with obesity of which 56% required ventilation.^9,11^

Referring to the protracted course of clinical presentation of COVID-19 and infection amongst obesity patients in the recent pandemic, patients with obesity should be carefully managed to avoid increased hospital stay, ICU admission and ventilation.^8^ An increase in non-COVID-19 mortality could be expected considering stringent lockdown regulations, fear of acquiring COVID-19 infection and reluctance of patients to seek medical care. Medical insurance companies and practitioners should consider promoting bariatric surgery to appropriate patients with obesity who fulfil the guidelines for bariatric-surgery-eligibility and who would benefit from bariatric surgery to decrease associated chronic comorbidities over a period of 12 months. This will alleviate the high risk for severe illness of any type of respiratory infections, especially COVID-19.
